# Structured diet and exercise guidance in pregnancy to improve health in women and their offspring: study protocol for the *Be Healthy in Pregnancy (BHIP)* randomized controlled trial

**DOI:** 10.1186/s13063-018-3065-x

**Published:** 2018-12-19

**Authors:** Maude Perreault, Stephanie A. Atkinson, Michelle F. Mottola, Stuart M. Phillips, Keyna Bracken, Eileen K. Hutton, Feng Xie, David Meyre, Rita E. Morassut, Harry Prapavessis, Lehana Thabane, the BHIP Study team, Stephanie A. Atkinson, Michelle F. Mottola, Keyna Bracken, Eileen K. Hutton, Lehana Thabane, Valerie Taylor, Olive Wahoush, Feng Xie, Stuart M. Phillips, Jennifer Vickers-Manzin, David Meyre, Harry Prapavessis

**Affiliations:** 1 Department Pediatrics, HSC 3A44, McMaster University, 1280 Main St W, Hamilton, ON L8S 4K1 Canada; 2 School of Kinesiology, Western University, London, ON Canada; 3 Department of Kinesiology, McMaster University, Hamilton, ON Canada; 4 Department of Family Medicine, McMaster University, Hamilton, ON Canada; 5 Department of Obstetrics & Gynecology, McMaster University, Hamilton, ON Canada; 6 Department of Health Research Methods, Evidence, and Impact, McMaster University, Hamilton, ON Canada; 7 Department of Pathology & Molecular Medicine, McMaster University, Hamilton, ON Canada

**Keywords:** Nutrition, Exercise, Randomized controlled trial, Pregnancy, Bone, Infancy, Developmental origins of health and disease, Proteins, Dairy foods, Gestational weight gain

## Abstract

**Background:**

Evidence from epidemiological and animal studies support the concept of programming fetal, neonatal, and adult health in response to in utero exposures such as maternal obesity and lifestyle variables. Excess gestational weight gain (GWG), maternal physical activity, and sub-optimal and excess nutrition during pregnancy may program the offspring’s risk of obesity. Maternal intake of dairy foods rich in high-quality proteins, calcium, and vitamin D may influence later bone health status. Current clinical practice guidelines for managing GWG are not founded on randomized trials and lack specific “active intervention ingredients.” The Be Healthy in Pregnancy (BHIP) study is a randomized controlled trial (RCT) designed to test the effectiveness of a novel structured and monitored Nutrition + Exercise intervention in pregnant women of all pre-pregnancy weight categories (except extreme obesity), delivered through prenatal care in community settings (rather than in hospital settings), on the likelihood of women achieving recommended GWG and a benefit to bone status of offspring and mother at birth and six months postpartum.

**Methods:**

The BHIP study is a two-site RCT that will recruit up to 242 participants aged > 18 years at 12–17 weeks of gestation. After baseline measures, participants are randomized to either a structured and monitored Nutrition + Exercise (intervention) or usual care (control) program for the duration of their pregnancy. The primary outcome of the study is the percent of women who achieve GWG within the Institute of Medicine (IOM) guidelines. The secondary outcomes include: (1) maternal bone status via blood bone biomarkers during pregnancy; (2) infant bone status in cord blood; (3) mother and infant bone status measured by dual-energy absorptiometry scanning (DXA scan) at six months postpartum; (4) other measures including maternal blood pressure, blood glucose and lipid profiles, % body fat, and postpartum weight retention; and (5) infant weight z-scores and fat mass at six months of age.

**Discussion:**

If effective, this RCT will generate high-quality evidence to refine the nutrition guidelines during pregnancy to improve the likelihood of women achieving recommended GWG. It will also demonstrate the importance of early nutrition on bone health in the offspring.

**Trial registration:**

ClinicalTrials.gov, NCT01689961 Registered on 21 September 2012.

**Electronic supplementary material:**

The online version of this article (10.1186/s13063-018-3065-x) contains supplementary material, which is available to authorized users.

## Background

### Consequences of excess gestational weight gain (GWG)

The documented adverse sequelae of excess GWG for both mother and child include undesirable pregnancy/birth outcomes and/or later health outcomes that are likely to impose a substantive downstream burden on healthcare costs [1]. The most common adverse effect of excessive GWG on maternal health is weight retention (five months and even up to three years postpartum) [2–4]. Mothers entering subsequent pregnancies at a higher weight are at greater risk for long-term obesity and future co-morbidities such as diabetes and cardiovascular disease [5, 6]. The emerging evidence of developmental origins of health and disease implicates the impact of mothers’ health during pregnancy on “programming” of the growing fetus in utero to develop adverse health outcomes [7, 8]. Maternal excess GWG has been reported as the strongest predictor of obesity in the offspring [9–11].

The use of low-fat dairy foods as the source of protein in pregnancy is proposed based on studies in animals [12] and humans [13–15] that provide support for the putative effects of components of dairy foods such as calcium and leucine as anti-adipogenic and pro-lipolytic agents. Consumption of high dairy protein may not only benefit GWG but also improve other maternal pregnancy outcomes such as dysglycemia, elevated blood pressure, blood lipid profiles, and risk of pre-eclampsia, the latter due to higher intake of calcium and vitamin D [16].

Guidelines for GWG exist and have been interpreted for practice in Canada [17, 18]. Until there is evidence of effective population level interventions to assist women in achieving healthy weights, programs aimed at interventions during pregnancy to improve weight management and pregnancy outcomes are a starting point. Pregnancy has been described as a “teachable moment” for weight control and obesity prevention in women [19]. The most convincing approach to achieve reduction in GWG is the combination of physical activity and diet counseling, preferably in combination with weight monitoring [1, 20, 21]. Yet current clinical practice guidelines for managing GWG are not founded on randomized trials and lack specific “active intervention ingredients” [21] that are proven effective in achieving target GWG.

### Maternal programming of bone health

Findings from epidemiological and animal studies support a role for the programming of fetal, neonatal, and adult bone outcomes in response to exposures during pregnancy such as maternal nutrition and other lifestyle variables [22–25]. In three longitudinal observational studies, maternal intake of dairy foods during pregnancy was associated with higher bone mass in offspring aged 6–16 years [26–28]. In prospective cohort studies, maternal physical activity level has also been positively associated with infant bone mass and geometry at birth [29, 30]. Being born with optimal bone mass may have long-term benefits since childhood bone mass tracks until skeletal maturity, when peak bone mass is achieved [31]. Collectively, these findings suggest that bone mass during childhood and adolescence may be predictive of an individual’s risk for osteoporosis, as peak bone mass is a strong predictor of osteoporotic fracture later in life [32].

To date, only one randomized controlled trial (RCT) in a small sample (*N* = 36) investigated the effect of dairy product supplementation on bone mineral density (BMD) and bone turn-over in pregnant women and these women entered pregnancy with a habitually low calcium intake [33]. The study showed a beneficial effect of increased milk consumption on maternal bone mass density at the spine and on suppression of bone resorption when measured at 6 ± 1 weeks postpartum. While promising, further studies are needed with larger sample sizes and evaluation of bone health outcomes in the offspring. In addition, no study to date has investigated the impact of dairy product supplementation during pregnancy on bone health outcomes of women after delivery.

Informed by our recent clinical and qualitative research, as well as existing systematic reviews, we have designed a RCT comprising several unique features with the objective of controlling GWG and optimizing bone health of mother and infant. We will test the effectiveness of a novel structured and monitored nutrition (high-protein dairy-based diet) along with an exercise (walking) intervention that is science-based, vetted for feasibility in pregnant women and care providers through focus groups, in pregnant women of most pre-pregnancy weight categories (except extreme obesity) in a community setting (as opposed to hospital-based).

### Specific objectives and hypothesis

The primary research objective of the Be Healthy in Pregnancy (BHIP) study is to determine whether introducing a structured and monitored nutrition (high-dairy protein diet) and exercise (walking) program (intervention) in early pregnancy, compared to standard prenatal care (control), will increase the number of women attaining GWG (outcome) within the Institute of Medicine (IOM) recommendations for their pre-pregnancy body mass index (BMI) sub-category [17].

Secondary objectives include determination of the impact of a maternal high-dairy diet with exercise compared to standard care diet during pregnancy on: (1) bone status (e.g. blood biomarkers procollagen type I that contains N-terminal extensions (PINP), C-terminal telopeptide of type I collagen (CTX-I), insulin-like growth factor-1 (IGF-1), 25-hydroxyvitamin D (25(OH)D), and 1,25-dihydroxyvitamin D) in mothers during pregnancy; (2) infant bone status at birth via cord blood; and (3) mother and infant bone status at six months postpartum by dual energy x-ray absorptiometry (DXA) scan.

The over-arching hypothesis is that maternal consumption of a high-dairy diet during pregnancy will have a positive impact on: (1) GWG; (2) bone health status of the mother during and after pregnancy; and (3) bone mass and bone size of the offspring, after adjustment for factors known to influence skeletal status.

## Methods/design

### Trial design

The BHIP study is a two-arm, two-site prospective RCT. This study is designed as a prospective superiority trial, with 1:1 allocation ratio to either the Intervention group (i.e. Nutrition + Exercise intervention: high-dairy nutrition intervention combined with structured exercise) or the Control group (i.e. usual care as per National Health Canada recommendations) during pregnancy. The study is open-label with blinded endpoints. The study protocol is described following the standard protocol items: recommendations for interventional trials (SPIRIT) guidelines [34]. See Additional file 1 for the SPIRIT checklist and Additional file 2 for the WHO trial registration data set.

The core BHIP trial follows women up to delivery as the primary outcome relates to GWG. The Bone-BHIP study is an extension of the core BHIP trial that continues a follow-up of the women and their offspring until six months after delivery, with the objective to assess maternal and infant bone status. This trial is registered at ClinicalTrial.gov (NCT01689961). The BHIP trial is conducted by collaborative research teams at McMaster University (Hamilton, ON) and Western University (London, ON) in Canada. The study takes place in academic hospitals and community healthcare clinics in London, Burlington, and Hamilton, Ontario, Canada. Recruitment is facilitated by healthcare professionals (e.g. family doctors and midwives) who ask their patients for consent to be contacted by the BHIP study team, as well as poster advertisements in midwifery, family practice, and ultrasound clinics, at community sites such as the YMCA, libraries, and coffee shops, and on Facebook or Kijiji.

**Fig. 1:**
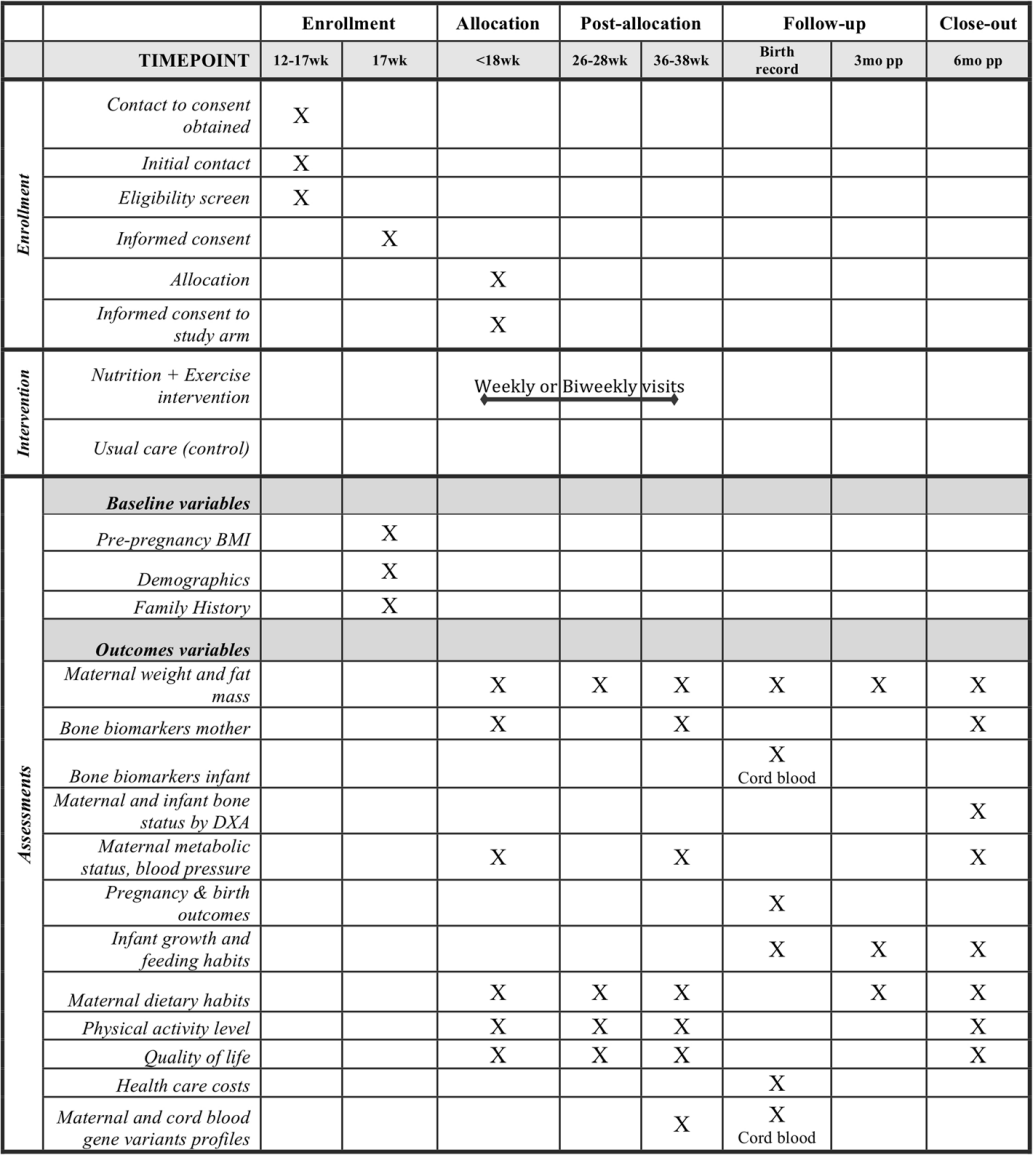
Flow diagram

Study recruitment began in January 2013 and is expected to be completed by April 2018, reaching the a priori calculated sample size of 242 participants for our primary outcome. All participants who sign consent to contact forms receive a phone call by study personnel and are provided a general overview of the study and expectations using a scripted text. Figure 1 outlines the participant’s timeline in the BHIP study. Participants are screened for eligibility during the telephone interview according to the criteria list below. Eligible women are enrolled in the study and sign informed consent by the end of the first trimester of pregnancy (i.e. 12–17 weeks of gestation).

#### Inclusion criteria

- Healthy pregnant women aged > 18 years
- Singleton pregnancy (either nulliparous or multiparous)
- Able to be randomized to group allocation by 17 weeks and six days of gestation
- Pre-pregnancy BMI < 40 kg/m^2^
- Planning to deliver at a Hamilton, Burlington, or London regional hospital or by home birth and willing to attend research visits at either study site
- Approval of primary care provider to participate in exercise
- Able to provide signed informed consent

#### Exclusion criteria

- Not conversant in English
- Known contraindications to exercise as recommended by the Canadian clinical practice guidelines for pregnancy [35]
- Severe chronic gastrointestinal, heart, kidney, liver, or pancreatic diseases or conditions
- Refusal to consume dairy foods due to intolerance or dislike
- Pre-existing diabetes
- Currently smoking and will not discontinue smoking during the pregnancy
- Depression score > 12 on the validated Edinburgh Depression scale

### Randomization: allocation and implementation

Block randomization is used with block sizes of two, four, and six selected at random, using an online Research Electronic Data Capture (REDCap) randomization service managed by an independent team in the Biostatistics Unit at St Joseph’s Healthcare in Hamilton, ON, Canada. Randomization to the two study arms occurs in a 1:1 ratio and is stratified by study site and pre-pregnancy BMI category following IOM guidelines (underweight: BMI < 18.5, normal weight: BMI 18.5–24.9, overweight: BMI 25.0–29.9, and obese: BMI ≥ 30.0 kg/m^2^). The eligibility of participants is confirmed at the first visit, when baseline data are also collected. The research assistant randomizes the participant at the second visit to the study center using an online third-party automatic randomization system. Once randomized, the research assistant consents the participant to the appropriate study arm at a time that is 14–17 weeks/6 days of gestation. Assessments, regardless of the study arm, occur at < 18 weeks of gestation, 26–28 weeks of gestation, 36–38 weeks of gestation, birth, three months postpartum, and six months postpartum. The intervention arm consists of weekly or biweekly in-person visits (based on the participant’s preferences) during pregnancy (i.e. from allocation to study group until 36–38 weeks of gestation). Baseline measures (i.e. before randomization), primary and secondary outcome measures, and other measures assessed during and after pregnancy are measured in all participants, regardless of their study group allocation.

**Fig. 2:**
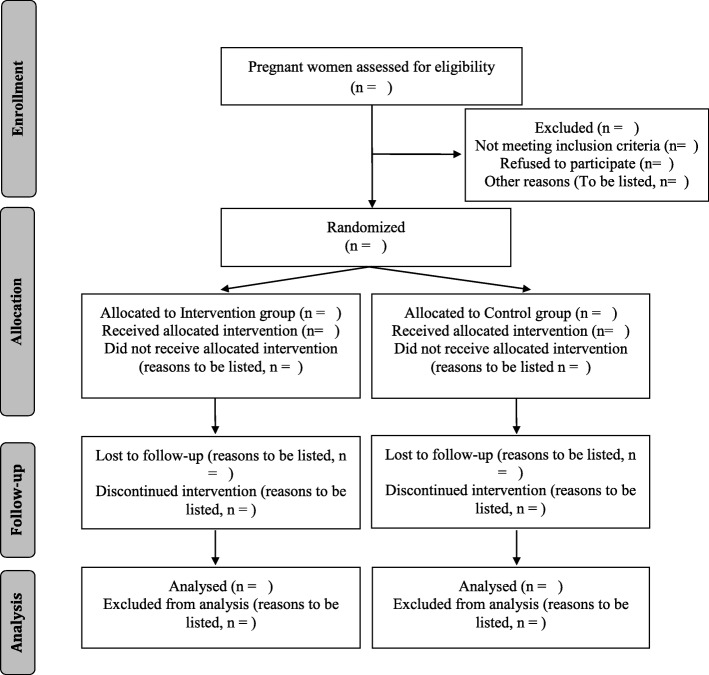
BHIP study protocol timeline

The flow diagram (Fig. 2) will be included in future published results.

This study is open-label due to the nature of the intervention. The study research assistants (data collectors) administering the personalized Nutrition + Exercise intervention are not blinded to group allocation. In addition, the participants are not blinded to their own group allocation, in order to maximize adherence to the lifestyle treatment. Mothers randomized to the intervention and control groups are assessed at the study clinic on different days, to prevent interaction between participants of each study arm. The primary data outcome collector is blinded to group allocation and is conducting the study visits for all participants, regardless of their group allocation. To maximize the objectivity of findings, the primary outcome assessor and data analysts/statistician are blinded to the study allocation and are not collecting any study data.

### Interventions

All participants receive usual care as delivered by their healthcare practitioner (family physician or midwife) during pregnancy. In addition, after enrolment in the RCT and regardless of their group allocation, all participants receive counseling by the study nutritionist on the latest recommendations by Health Canada as published online (https://www.canada.ca/en/health-canada/services/healthy-living/healthy-pregnancy.html). After delivery, both study groups receive usual care from their healthcare practitioners with no further intervention; the research team prospectively follows participants at three months and six months postpartum. Table 1 outlines the study arms.

**Table 1:** BHIP study arms

Component	Control arm *Usual care* 12–17 to 36–38 weeks of gestation	Intervention arm *Nutrition + Exercise intervention* 12–17 to 36–38 weeks of gestation
Gestational weight gain (GWG)	Latest recommendation by Health Canada in terms of GWG [17] and the Pregnancy Weight Gain Calculator [44]	Latest recommendation by Health Canada in terms of GWG [17]
Nutrition during pregnancy	Latest recommendation by Health Canada in terms of nutrition during pregnancy [74] and Eating Well with Canada’s Food Guide [46]	Latest recommendation by Health Canada in terms of nutrition during pregnancy [74] **Nutrition component of the intervention**
		1. Individualized nutrition plan with a high protein content: ~ 25% of energy intake which is within the acceptable macronutrient distribution range a. Individualized to each mother’s estimated energy requirement and calculated using the equation derived in the Dietary Reference Intake report for women [43] with energy intakes adjusted as recommended in the second and third trimesters [74] b. Provided by dairy foods: 4–5 servings of dairy food/d: fresh low-fat white milk and/or cottage cheese and/or low-fat Greek yogurt, as implemented in our previous study [38]
Exercise during pregnancy	Latest recommendation by Health Canada in terms of exercise during pregnancy [75]	Latest recommendation by Health Canada in terms of exercise during pregnancy [75] **Exercise component of the intervention**
		1. Controlled walking program with the study nutritionist a. 25 min per session, 3–4 times per week, while increasing the time by 2 min/week to a maximum of 40 min maintained until delivery [39] 2. 10,000 steps per day a. Daily step counts and any other exercise tracked using a pedometer every day and an exercise log. Also used as a motivator and self-monitoring device which will also help improve compliance [39]
Wellbeing during pregnancy	**Nested qualitative study**	
	1. Focus group and information session given by a midwife in the third trimester of pregnancy a. Discussion on topics such as pain relief options during labor and breastfeeding techniques	

Participants randomized to the personalized, monitored, and structured Nutrition + Exercise program group (intervention) begin early in the second trimester of their pregnancy until the end of pregnancy with counseling on a weekly or biweekly basis (Table 1 and Fig. 1). The nutrition component is an individualized nutrition plan tailored to each participant’s energy requirements with a high protein content (25% protein energy) provided primarily by dairy foods. Dairy foods are accepted by women during pregnancy as a healthy choice as indicated from our pilot study [36] and in a recent birth cohort study in the same community in which women consumed an average of ≥ 3 servings of dairy foods per day [37]. Dairy foods are sources of high-quality proteins, calcium, and vitamin D in the case of milk as it is under mandatory fortification with vitamin D in Canada. The Nutrition + Exercise intervention is considered safe. First, the nutrient intake associated with the increased low-fat dairy consumption is less than the tolerable upper intake level for all nutrients as recommended per the Dietary Reference Intake by Health Canada. In addition, walking (the exercise component) is the easiest physical activity to undertake and implement in terms of goal-setting for step count and monitoring adherence using accelerometer-type devices as demonstrated in our previous studies [38–40]. Walking was the most practical exercise intervention of choice since women reduce moderate and vigorous physical activity during pregnancy yet maintain levels of walking [41]. Most importantly, these exercise guidelines are based on the Physical Activity Readiness Medical Examination (PARmed-X) for Pregnancy [35]. Data from the Nutrition and Exercise Lifestyle Intervention Program study [39] indicates that the goal of 10,000 daily steps is feasible under free-living conditions and has been effectively utilized in a number of trials [42].

A component of the intervention is discontinued if the medical condition of a participant changes and the intervention is modified if a participant requests a change due to personal reasons, such as food aversion or pain episode. The intervention is personalized for every woman, to ensure the goal of 25% energy from protein and 10,000 steps/day are achieved. Additionally, estimated energy requirements are calculated for each mother using the equation derived in the Dietary Reference Intake report for normal and overweight women [43] with energy intakes adjusted as recommended in the second and third trimesters [17]. The individualized nutrition plan is fashioned upon a standard three-day food intake record completed by the mothers that includes one weekend day as used previously in pregnant women [39]. The individualized nutrition plans and counseling are conducted by a study nutritionist providing the number of food servings to meet their estimated energy requirements. The nutrition plan is modified if GWG monitoring indicates weight loss or excess gain has occurred as recommended in the GWG guidelines [17, 44]. Adverse events related to the intervention are reported to our local ethics boards. Other non-intervention-related adverse events are noted in the participant’s chart and monitored, in accordance with the N2 Network of Networks guidelines Canada [45].

Several strategies are used to improve adherence to the intervention. For the exercise component, participants wear and track their step counts on a weekly basis, increasing motivation and adherence to the treatment. In addition, they go for a walk with the research staff and have a personalized counseling session with the study nutritionist. For the nutrition component, participants are provided with low-fat milk, cottage cheese, and/or yogurt, as per their preference, to ensure they consume the recommended amount of dairy foods. Participants come to the study site at least every other week to receive their dairy foods and the study nutritionist shares strategies and recipes with the participants to ensure they consume the targeted amount of dairy food servings. All these actions improved the adherence to the study protocol.

Participants randomized to the usual care group (control) receive usual prenatal care. Participants are given the most recent advice from Health Canada including Healthy Weight Gain During Pregnancy [17], the Pregnancy Weight Gain Calculator [44], and Eating Well with Canada’s Food Guide [46]. Mothers are followed by their primary care provider who also receives the same Health Canada materials. After delivery, they are followed by their healthcare practitioners. In addition, the control participants are invited to participate in a focus group and to attend an information session led by a midwife; topics discussed include pain relief options during labor and breastfeeding techniques (Table 1).

## Outcome measurements

Primary and secondary outcomes are summarized in Table 2 and described below.

- Primary outcome
  - ○ GWG
- Secondary outcomes
  - ○ Maternal and cord blood circulating bone markers: PINP, CTX-I, IGF-1, 25(OH)D, 1,25dihydroxyvitamin D
  - ○ Maternal bone status at six months postpartum: whole-body bone mineral content (BMC), whole body BMD, lumbar spine BMD
  - ○ Infant bone status at six months of age: whole body minus the head BMC
- Other outcomes
  - ○ Maternal body weight and fat mass
  - ○ Maternal metabolic status (including fasting glucose and lipid profiles, leptin and adiponectin)
  - ○ Maternal blood pressure
  - ○ Maternal pregnancy outcomes such as gestational diabetes and pre-eclampsia
  - ○ Infant birth outcomes such as birth weight and body fat mass at six months
  - ○ Safety outcomes
  - ○ Quality of life using the EQ-5D questionnaire
  - ○ Healthcare costs
  - ○ Maternal and cord blood gene variant profiles

**Table 2:** Analysis plan: objectives, outcomes, hypotheses, and methods of analysis

Objective	Hypothesis	Outcome measure (type of outcome: B = binary or C = continuous)	Methods of analysis
1. Primary	An experimental combined Nutrition + Exercise intervention will increase the percentage of pregnant women who achieve GWG within current recommendations when compared with standard care provided in the primary care community setting	Proportion of women who are within the BMI appropriate GWG according to the IOM guideline for GWGs (B)	Logistic regression
2. Secondary	An experimental combined Nutrition + Exercise intervention will lead to better maternal and child bone health outcomes when compared to standard care	*• Maternal and cord blood circulating bone markers (C)* ○ Bone biomarkers: PINP, CTX-I, IGF-1, 25(OH)D, 1,25(OH)_2_D *• Maternal bone status at 6 months postpartum (C)* ○ Whole body bone mineral content, whole body BMD, lumbar spine bone mineral density by DXA scan *• Maternal fat mass (C)* *• Maternal blood glucose, lipid profile, leptin, and adiponectin* *• Maternal blood pressure (C)* ○ Diastolic BP ○ Systolic BP *• Maternal pregnancy outcomes (B)* ○ Gestational diabetes ○ Pre-eclampsia *• Infant bone status at 6 months of age (C)* ○ Whole body minus the head bone mineral content by DXA scan *• Infant outcomes* ○ Birth weight z-score(C) ○ Body fat mass (B)	Regression analysis *We will use logistic regression for binary outcomes and linear regression for continuous outcomes
3. Subgroup analyses	The percentage of women within each of the normal, overweight, and obese BMI categories will be similar with respect to being with the IOM target GWG for each category	Proportion of women in each BMI category who reach appropriate GWG according to the IOM guideline for GWGs	Regression analysis including the interaction term of BMI group X Intervention group
4. Sensitivity analyses	Combined Nutrition + Exercise Intervention leads to a greater percentage of women who achieve GWG within current recommendations when compared to standard care	Primary outcome only	***•*** Generalized estimating equations ***•*** Random-effects model

IMPORTANT REMARKS:

In all analyses, results will be expressed as difference or OR (95% CI) and associated *p* values, as appropriate

Bonferroni method will be used to adjust the overall level of significance for multiple secondary outcomes

We will examine residuals to assess model assumptions

The GEE [76] is a technique that allows to specify the correlation structure between patients within a site and this approach produces unbiased estimates under the assumption that missing observations will be missing at random. An amended approach of weighted GEE will be employed if missingness is found not to be at random [77]

*Infant growth outcomes at 6 months will be adjusted for feeding type (duration of breast feeding from birth to 6 months)

### Data and biosample collection

The pre-pregnancy BMI is calculated using the measured weight at study entry minus the mother’s self-reported GWG. Body weight is measured by trained research assistants using a body impedance Tanita Body Composition Analyzer BF-350 scale (Tanita, IL, USA) at 12–17 weeks of gestation, 26–28 weeks of gestation, 36–38 weeks of gestation, three months postpartum (self-reported weight only), and six months postpartum. GWG is calculated by subtracting the weight at the 36–38 weeks of gestation study visit by the pre-pregnancy weight. Adjustments such as rate of GWG will be made to account for gestational age at delivery, including pre-term births.

Fasted maternal blood samples for metabolic and bone health biomarkers are collected at 12–17 weeks of gestation, 36–38 weeks of gestation, and at six months postpartum; venous cord blood is collected at delivery. Serum is collected in serum-separating with gel (SST™) vacutainers and in spray-coated silica vacutainers; plasma is collected in sodium fluoride/ Na_2_ ethylenediaminetetraacetic acid (Na2EDTA) vacutainers. Sample are centrifuged for 10 min at 3000 rpm at 4 °C; serum separator tubes are spun for an additional 5 min, aliquoted, and stored at − 80 °C. Samples are batched to include all samples from each mother for analysis in our laboratories when participants have completed the study. Fasting plasma glucose is determined using a hexokinase photometric assay (Architect kit, Abbott, Abbott Park, IL, USA). Serum triglycerides are analyzed using a glycerol phosphate oxidase photometric assay (Architect kit, Abbott, Abbott Park, IL, USA) completed by Hamilton Health Sciences Regional Laboratory Medicine Program. Serum leptin and insulin are analyzed using Luminex® human premixed multi-analyte enzyme-linked immunosorbent assay (ELISA) kit supplied by R&D Systems (Minneapolis, MN, USA). Serum adiponectin and C-reactive protein are analyzed by Luminex® premixed multi-analyte ELISA kit supplied by R&D Systems. Bone formation is assessed by measuring serum PINP by ELISA (Cloud Clone Corp., Houston, TX, USA). Bone resorption is assessed by measuring serum CTX-I by (Serum Crosslaps (CTX-1) ELISA, product code AC-02F1 Immunodiagnostic Systems, UK). Growth is assessed by measuring serum IGF-1 by ELISA (R&D Systems, Minneapolis, MN, USA). All standards and samples are analyzed in duplicate, following the manufacturer’s instructions. A serum quality control is run in triplicate at the beginning and end of each ELISA plate to calculate inter- and intra-assay coefficients of variability. Serum vitamin D metabolites are measured by liquid chromatography tandem mass spectrometry (LC-MS/MS) using a Waters AQUITY Tandem Quadropole Detector coupled to an AQUITY UPLC system (Waters Corporation), after extraction that includes a saponification step as adapted from Hymoller [47]. Accuracy is determined using the certified Vitamin D analytes serum-based standard reference material (SRM 972a) from the US National Institute for Standards and Technology (NIST; National Bureau of Standards, Washington, DC, USA) [48]. Precision is measured using a serum quality control in all runs of samples tested, allowing calculation of inter- and intra-assay coefficients of variability.

DNA extracted from maternal and cord blood plasma samples will be purified using the Chemagen 500 MSM I (PerkinElmer chemagen Technologie GmbH, Baesweiler, Germany) and genotyped using the Human Core Exome Bead Chip by Illumina. The chip is specifically designed to allow imputation of up to 18 million markers in multi-ethnic populations. Data cleaning and quality control of SNPs data are done according to the guidelines published by Winkler et al. [49].

Bone mass is measured by DXA scan using the research dedicated QDR®4500 series Hologic Inc. Discovery™ DXA machine (Waltham, MA, USA; Adult whole body software version 12.3.1 and Infant whole body software) at the McMaster University study site and the General Electric-Luna iDXA (Ames Medical enCORE, Version 14.1, Waukesha, WI, USA; CoreScan, GE) at the Western University study site. Daily quality control tests are conducted using an artificial L_1–4_ lumbar spine made from hydroxyapatite encased in epoxyresin. Weekly, a calibration test is performed using a step phantom (composed of soft-tissue and lean-tissue equivalent materials); at this time, the uniformity test is also performed to evaluate the contribution of air molecules to the attenuation of the X-rays. The coefficient of variations for BMC and BMD were 0.65% and 0.38%, respectively (Hologic Inc., Bedford, MA, USA) in our past study [37]. Inter-site comparison of DXA measures is conducted by having two phantoms (i.e. Hologic lumbar spine phantom (#2603), BFP® phantom) tested at both study sites. This provides a cross-calibration standard and enables us to account for inter-site differences and to harmonize results. It also allows for verification of the instrument stability over the course of the study.

At six months postpartum, bone scans are performed on mothers and infants at six months by staff following a standard operating procedure. Mothers are dressed in regular clothes without any metal parts or in a light hospital gown, while infants are in a clean diaper only. The primary outcome assessor, who has expertise in analyzing DXA scans, reviews all scans from both study sites. This ensures consistency in analysis across all participants at both study sites. If objects are included in the scan such as a child’s toy, a sub-regional analysis is done to isolate and subtract the object’s contribution to overall results. If considerable movement artifacts are seen in infant scans, the well-captured limb is used as a surrogate for the one with movement [50]. Any scans with unsalvageable distortions due to movement are not included. Z-scores are calculated for all BMC and BMD results using data from an age- and sex-specific standard curve. Results for women are interpreted as z-scores in reference to an adult women population embedded in the Hologic software. Results for infants are expressed as whole body minus the head BMC and BMD. Data are interpreted as z-scores in reference to an infant population from in-house data collection on normal healthy term infants from birth to one year of age.

All participants receive instructions to complete a standard three-day food intake record at 12–17 weeks of gestation, 26–28 weeks of gestation, 36–38 weeks of gestation, and six months postpartum. The food record includes two weekdays and one weekend day, as used previously in pregnant women [39]. It captures food and beverage intake as well as drug and supplement (vitamin and mineral) intake. Trained assessors analyze all food records using standard operating procedure on the computer-based Nutritionist Pro Software (Axxya Systems, Stafford, TX, USA). Nutritionist Pro computes the average amount of nutrients consumed per day.

Physical activity level is assessed through wearing a BodyMedia Sensewear Pro II armband monitor device (BodyMedia, Pittsburgh, PA, USA) in all participants for the same three days (as food monitoring above) in their work/home environment. These small devices are comfortable and easily wearable on the arm [38]. The data collected for three days at each visit (12–17, 26–28, and 36–38 weeks of gestation and six months postpartum) includes daily steps, daily energy expenditure, and minutes of activity level in Metabolic Equivalent of Task (MET; very vigorous, vigorous, moderate, sedentary). The Sensewear unit does not provide feedback to the participant.

Pregnancy and infant outcomes are obtained from participant’s medical records. Details are extracted about Cesarean section and/or vaginal delivery. For infant outcomes, data regarding gestational age, birth weight, birth length, birth head circumference, 1-min and 5-min Apgar scores, complications related to birth, and feeding practices at birth are extracted.

Infant weight and length at birth are recorded from medical charts and at three months of age as reported by the mother during the telephone interview. At the six-month visit, trained assessors measure infant weight by electronic scale (Medela BabyWeigh, McHenry, IL, USA), length using a measuring board for term infants (Pediatric Stadiometer, Ellard Instrumentation Ltd., Monroe, WA, USA), and head circumference using a constant-tension measuring tape (OHAUS, Dundas, ON, Canada), following standard operating procedures.

Infant feeding practices over the first three months are recorded by phone call and at six months in person using a standardized questionnaire administered by a trained assessor [51]. The questionnaire includes feeding history, duration and extent of breastfeeding, introduction of solid foods, and use of vitamins and supplements.

### Study management and governance

This RCT is led by investigators from McMaster University (Department of Pediatrics, Department of Family Medicine, Department of Obstetrics and Gynecology, Department of Health Research Methods, Evidence and Impact, and School of Nursing) and Western University (School of Kinesiology) as well as practitioners from the City of Hamilton Public Health, all located in Ontario, Canada. The oversight of the study is guided by the steering committee that is composed of the lead investigators (SAA, SMP, MFM, LT, and EKH). The sponsors of the study are McMaster University and Western University. The sponsors are indemnified for any harms arising from trial participation and they approve protocol amendments when ethics approval has been obtained. Day-to-day running of the study is provided by the trial principal investigators, the study coordinators, and research assistants. The monitoring board consists of three well-established investigators from universities within Canada but outside McMaster and Western Universities.

All case report forms are anonymized using study identifiers and are stored in locked cabinets in a locked office. An administrative assistant to the study team who is not involved in the study holds the key to participants’ names. Research staff who are not involved in data collection enter data in a two-step process (entry and verification, following standard operating procedures) in our REDCap projects hosted at McMaster University [52]. REDCap is a secure, web-based application designed to support data capture for research studies, providing: (1) an intuitive interface for validated data entry; (2) audit trails for tracking data manipulation and export procedures; (3) automated export procedures for seamless data downloads to common statistical packages; and (4) procedures for importing data from external sources. Use of range check for data values promotes data quality. Double data entry is performed to ensure inter-study site reliability.

The research assistant monitors intervention adherence biweekly. For the nutrition portion, this includes ensuring dietary intake meets each participant’s caloric needs and are within an acceptable macronutrient distribution range. In terms of the exercise portion, participants monitor and report biweekly their physical activity using a diary and by wearing a pedometer. Participants report any discomfort experienced (due to the natural course of pregnancy and/or the intervention) to the study coordinator who personalizes the intervention as needed.

### Statistical methods

The analysis and reporting of the results will follow the SPIRIT statement for reporting RCTs [53]. The process of patient selection and flow throughout the study will be summarized using a flow-diagram (Fig. 2).

The results of patient baseline characteristics and outcome variables (both primary and secondary) will be summarized using descriptive summary measures: expressed as mean (standard deviation) or median (range) for continuous variables and n (%) for categorical variables. All statistical tests will be performed using two-sided tests at the 0.05 level of significance. Binary outcomes will be analyzed using logistic regression and continuous outcomes will be analyzed using linear regression. All analyses will account for stratification by including study site and participants’ BMI category in the models. For all models, the results will be expressed as estimate of the difference for continuous outcomes (odds ratio [OR] or relative risk [RR] for binary outcomes), corresponding two-sided 95% confidence intervals (CI) and associated *p* values. *P* values will be reported to three decimal places with values < 0.001 reported as < 0.001. The Hosmer–Lemshow will be used to assess goodness-of-fit for logistic regression and explore the residuals to assess goodness-of-fit and model assumptions for linear regression.

An intention-to-treat principle will be adopted to analyze all outcomes. Multiple-imputation will also be used to handle missing data during data collection. Sensitivity analyses will be performed using some of the commonly used RCT patient-level methods (e.g. generalized estimating equations, the random-effect model which account for the potential correlation within sites, etc.) to assess the robustness of the results [54]. All the analyses will be done using SAS 9.2 (Cary, NC, USA) or SPSS 13 (Chicago, IL, USA). Table 2 provides a summary of the planned methods of analyses for each outcome and subgroup.

### Power calculation

The sample size calculation for the core study is based on the test of the null hypothesis that the percentages of women with GWG within IOM guidelines in the two populations (intervention and control) are equal. To account for the uncertainty in these prior estimates, we calculated the sample size for different values of the percentage of women with weight within IOM guidelines in the control group in the range of 50–65%, with a risk difference of 15%, 20%, 25%, and 30%. While initially we targeted a total sample size of 350, the constraints of our funding timelines (which include a one-year CHIR grant extension) and a recruitment rate that was less than projected despite our best efforts, we revised our desired sample size to 242. Based on our a priori calculation, a sample size 111 per group corresponds to an absolute difference of 30% with our Nutrition + Exercise intervention, resulting in 30% of women in the treatment group having a GWG exceeding the IOM recommendations compared to 65% in the control group. With the revised sample size (i.e. assuming a 1:1 allocation ratio), the study will have power of 80% to yield a statistically significant result assuming a binomial distribution (using an intention-to-treat principle for the analysis) of the difference between percentages of women with GWG within IOM guidelines at alpha = 0.05. We hope to increase the sample size to 242 total participants in order to allow us to account for the two stratification variables – site (2 degrees of freedom) and baseline BMI strata (3 degrees of freedom).

The calculated sample size for the secondary outcome of maternal bone biomarker status is a total of 177 participants (allocation 1:1, α = 0.05, β = 20 for two-group t-test), based on literature with similar outcomes [55, 56]. A sample size was also calculated for the secondary outcome of infant whole body minus the head BMD. The total is 240 participants (allocation 1:1, α = 0.05, β = 20 for two-group t-test) based on the literature with similar primary outcomes in infant populations [57, 58]. For both sample sizes, a 15% attrition rate was calculated as a precaution in this type of clinical trial with follow-up.

## Discussion

The BHIP study is novel as it proposes a feasible Nutrition + Exercise intervention that pregnant women could incorporate into their lifestyle and improve adherence to GWG recommendations. In addition, it will contribute to the knowledge of the perinatal developmental programming of body composition and skeletal phenotypes.

The BHIP RCT enrolls women in early pregnancy (12–17 weeks of gestation). Ideally, women should have access to general health interventions before pregnancy; however, currently pregnancy is often the first opportunity for health professionals to address issues beyond those directly related to the pregnancy state. Thus, one of the strengths of the BHIP study is that it starts in early pregnancy and monitors participants until six months postpartum. In addition, measures of health outcomes in both mother and child at various time points throughout pregnancy and postpartum allow for a comprehensive evaluation of the intervention effects. Lastly, one particular strength of the BHIP study is the individualized intervention and weekly monitoring. Evidence shows that the consumption of dairy foods (nutrition component) is well accepted during pregnancy [36], while walking (exercise component) is the best physical activity to promote adherence during pregnancy [38–40].

The primary outcome of GWG was selected because it is the key clinical problem we are addressing with our Nutrition + Exercise intervention. Pre-pregnancy obesity and excess GWG are the strongest maternal characteristics associated with offspring obesity [9, 11, 59] and childhood metabolic dysfunction [60]. Excess GWG in pregnancy is a major clinical challenge affecting Canadian women who enter pregnancy overweight and even women of normal pre-pregnancy weight [61, 62]. At the national level, a cross-sectional study on pregnancy experiences in Canada determined that over one-third of women entered pregnancy overweight or obese and nearly 60% experienced GWG greater than that recommended by the IOM recommendations for GWG [63]. The Alberta Pregnancy Outcomes and Nutrition prospective cohort study reported similar statistics with a greater number of overweight and obese women (80%) gaining excess weight during pregnancy [64]. Likewise, in the Hamilton region, data from the Family Atherosclerosis Monitoring In earLY life birth cohort demonstrated that > 50% of women entered pregnancy with a pre-pregnancy BMI of > 25.0 kg/m^2^ and > 50% exceeded the IOM guidelines for GWG [65]. Evidence to date suggest that the most convincing approach to achieve appropriate GWG is an intervention combining physical activity and nutrition, in combination with weight monitoring [1, 20, 21].

The secondary outcome of bone status was selected due to the emerging evidence of risk of osteoporosis in later life being programmed from the womb [24]. Bone biomarkers were selected as outcomes following the International Osteoporosis Foundation and the International Federation of Clinical Chemistry and Laboratory Medicine recommendations, which is to measure a marker of bone formation and a marker of bone resorption to assess bone turnover [66]. Currently, the most sensitive markers recommended are subgroups of type I collagen representing the predominant component of the bone matrix wherein sub-products can be measured as markers of formation and resorption of the bone [66]. To support fetal bone growth, pregnant women undergo bone metabolic adaptations over the course of pregnancy, where bone resorption usually rises and formation declines [67]. These changes result in high bone turnover during pregnancy [68]. Maternal bone turnover peaks during the third trimester, with a marked increase in bone resorption observed at 38 weeks of gestation [69]. Based on this knowledge, two biomarkers of bone modelling are quantified in maternal blood at 36–38 weeks of gestation and in cord blood. The marker of bone formation is PINP and the marker of bone resorption is CTX-I. Blood samples are consistently collected in a fasted state and in the morning, as suggested by the National Bone Health Alliance [70]. In addition, vitamin D status is measured by serum 25(OH)D as it plays an important role in calcium homeostasis and bone metabolism [71] and is a reflection of dietary vitamin D intake and sun exposure. Both the frequency of milk consumption and sun exposure were shown to be positive predictors of serum 25(OH)D concentrations in the recent multivariate analysis of three cycles of the Canadian Community Health Measures surveys [72]. The active form of vitamin D, 1,25dihydroxyvitamin D, is also measured since it is known to be upregulated during pregnancy [73].

Bone mineral content was selected as an outcome because it is linked to the peak bone mass (i.e. highest bone mass accrued for an individual) and is predictive of osteoporotic fracture [32]. Furthermore, recent evidence suggests that childhood BMC persists until peak bone mass and could indicate individuals at risk of osteoporotic fracture [31].

The nature of the intervention brings a limitation; the research study personnel in direct contact with the participants are not blinded to the study allocation of each participant. To preserve the integrity of the study findings and the internal validity of the study, all other investigators and statisticians are blinded to the study allocation, including people collecting and performing the analysis of primary and secondary outcomes. Some sources of bias are inevitable in the context of the study: recruitment sites are mostly primary care clinics, with few participants recruited from advertisements in the community. Since women are excluded with limited comprehension of the English language, if documented signs of depression or a pre-pregnancy BMI > 40 kg/m^2^, the generalization of the study would exclude such groups. From previous studies completed in urban Southern Ontario [39, 65] we anticipate that our study population will be mostly Caucasian, holding a university degree, and with a medium to high socioeconomic status. This sample is representative of the populations in the cities were recruitment occurred as they are modern urban centers with universities, colleges, and major commerce. Thus, the results will be generalizable to populations of women living in many Canadian cities with academic centers and major commerce. In addition, our results will still be of high value for the general Canadian pregnant women population who are served through provincial public health units, as it will bring new knowledge to refine the Canada Prenatal Nutrition Program (CPNP, https://www.canada.ca/en/public-health/services/health-promotion/childhood-adolescence/programs-initiatives/canada-prenatal-nutrition-program-cpnp.html), which is currently offered through public health but not based on evidence.

The strengths of the design include that the Nutrition + Exercise intervention was vetted for feasibility in pregnant women and care providers through focus groups and was conducted in pregnant women of all pre-pregnancy BMI categories (except extreme obesity) and in a community setting (as opposed to hospital-based as in many reported studies).

For the timely recruitment of study participants, challenges included prolonged ethics approval processes at secondary recruitment sites, reduced recruitment at community family practices due to influx of immigrants who were non-English speaking (thus not eligible), and competition with other studies sampling pregnant women. In light of these, we have taken measures to achieve the predetermined sample size based on a power analysis for the primary outcome so that the integrity of the study is preserved and the results are able to properly test the stated hypothesis and provide the needed new knowledge. To expand recruitment at McMaster University a new local study site was set up in a nearby city, Burlington, Ontario, with the collaboration of midwifery and obstetric clinics, as well as the local hospital. This partnership is very successful thus helping to achieve the recruitment goal. To date, retention after randomization to the study visit at 36–38 week of gestation when we assessed our primary outcome is 87%. To maximize retention of participants, we implemented a number of procedures such as study visit times at the convenience of the participants, including home visits. We also offered in-person, over the phone, and email correspondence to keep participants engaged. For participants in the intervention group, providing them with the dairy products (which are an integral part of the intervention) increases the likelihood of consuming the recommended amount. For the exercise component, participants receive a free pedometer as a self-motivation tool to reach their step count goals. Lastly, for both groups, participants are compensated on three occasions with $25 gift cards that they can redeem at a local grocery store.

If effective, this RCT will generate high-quality evidence to refine the nutrition guidelines during pregnancy to improve health of women during pregnancy and their offspring in early life, including acquisition of a strong skeleton.

## Trial status

Active recruitment continues since a no-cost extension of one year from CIHR was obtained in December 2016, to allow the BHP study to achieve the target sample size.

## Additional file

Additional file 1: SPIRIT checklist. (DOCX 76 kb)

Additional file 2: WHO Trial registration data set. (DOCX 18 kb)

## Abbreviations

25(OH)D: 25-hydroxyvitamin D
AAFC: Agriculture and Agri-Food Canada
BHIP study: Be Healthy in Pregnancy study
BMC: Bone mineral content
BMD: Bone mineral density
BMI: Body mass index
CIHR: Canadian Institutes of Health Research
CTX-I: C-terminal telopeptide of type I collagen
DXA: Dual-energy X-ray absorptiometry scan
ELISA: Enzyme-linked immunosorbent assay
GWG: Gestational weight gain
IGF-1: Insulin-like growth factor-1
IOM: Institute of Medicine
LC-MS/MS: Liquid chromatography tandem mass spectrometry
MET: Metabolic Equivalent of Task
NIST: US National Institute for Standards and Technology
OR: Odds ratio
PARmed-X: Physical Activity Readiness Medical Examination for Pregnancy
PINP: Procollagen type I that contains N-terminal extensions
RCT: Randomized controlled trial
REDCap: Research Electronic Data Capture
RR: Relative risk
SPIRIT: Standard protocol items: recommendations for interventional trials
SST: Serum-separating vacutainers

## References

[1] Quinlivan JA, Lam LT, Fisher J (2011). A randomised trial of a four-step multidisciplinary approach to the antenatal care of obese pregnant women. Aust New Zeal J Obstet Gynaecol.

[2] Viswanathan M, Siega-Riz AM, Moos MK, Deierlein A, Mumford S, Knaack J, et al. Outcomes of maternal weight gain. Evid Rep Technol Assess (Full Rep). 2008;3(168):1–223. PMC4781425 18620471

[3] Vasudevan C, Renfrew M, McGuire W (2011). Fetal and perinatal consequences of maternal obesity. Arch Dis Child Fetal Neonatal Ed.

[4] Thornton YS, Smarkola C, Kopacz SM, Ishoof SB (2009). Perinatal outcomes in nutritionally monitored obese pregnant women: a randomized clinical trial. J Natl Med Assoc.

[5] Nohr E, Vaeth M, Baker J, Sørensen T, Olsen J, Rasmussen K (2009). Pregnancy outcomes related to gestational weight gain in women defined by their body mass index, parity, height, and smoking status. Am J Clin Nutr.

[6] Poston L, Harthoorn LF, Van Der Beek EM (2011). Obesity in pregnancy: Implications for the mother and lifelong health of the child. A consensus statement. Pediatr Res.

[7] McDonald SD, Han Z, Mulla S, Beyene J (2010). Overweight and obesity in mothers and risk of preterm birth and low birth weight infants: systematic review and meta-analyses. BMJ.

[8] Fraser A, Tilling K, MacDonald-Wallis C, Sattar N, Brion MJ, Benfield L (2010). Association of maternal weight gain in pregnancy with offspring obesity and metabolic and vascular traits in childhood. Circulation.

[9] Eriksson JG, Sandboge S, Salonen M, Kajantie E, Osmond C (2015). Maternal weight in pregnancy and offspring body composition in late adulthood: Findings from the Helsinki Birth Cohort Study (HBCS). Ann Med.

[10] Gillman MW, Rifas-Shiman SL, Kleinman K, Oken E, Rich-Edwards JW, Taveras EM (2008). Developmental origins of childhood overweight: potential public health impact. Obesity.

[11] Morandi A, Meyre D, Lobbens S, Kleinman K, Kaakinen M, Rifas-Shiman SL (2012). Estimation of newborn risk for child or adolescent obesity: lessons from longitudinal birth cohorts. PLoS One.

[12] Thomas AP, Dunn TN, Drayton JB, Oort PJ, Adams SH (2012). A high calcium diet containing nonfat dry milk reduces weight gain and associated adipose tissue inflammation in diet-induced obese mice when compared to high calcium alone. Nutr Metab.

[13] Zemel MB (2009). Proposed role of calcium and dairy food components in weight management and metabolic health. Phys Sportsmed.

[14] Zemel MB, Donnelly JE, Smith BK, Sullivan DK, Richards J, Morgan-Hanusa D (2008). Effects of dairy intake on weight maintenance. Nutr Metab.

[15] Teegarden D, White KM, Lyle RM, Zemel MB, Van Loan MD, Matkovic V (2008). Calcium and dairy product modulation of lipid utilization and energy expenditure. Obesity.

[16] Robinson CJ, Alanis MC, Wagner CL, Hollis BW, Johnson DD (2010). Plasma 25-hydroxyvitamin D levels in early-onset severe preeclampsia. Am J Obstet Gynecol.

[17] Health Canada. Prenatal Nutrition Guidelines for Health Professionals, Gestational Weight Gain. 2010. https://www.canada.ca/en/health-canada/services/food-nutrition/healthy-eating/prenatal-nutrition/eating-well-being-active-towards-healthy-weight-gain-pregnancy-2010.html. Accessed 4 Dec 2018.

[18] Institute of Medicine. Re-examination of IOM Pregnancy Weight Guidelines 2009. 2011. https://www.ncbi.nlm.nih.gov/books/NBK32813/. Accessed 4 Dec 2018.

[19] Phelan S (2010). Pregnancy: A “teachable moment” for weight control and obesity prevention. Am J Obs Gynecol.

[20] Dodd JM, Grivell RM, Crowther CA, Robinson JS (2010). Antenatal interventions for overweight or obese pregnant women: A systematic review of randomised trials. BJOG An Int J Obstet Gynaecol.

[21] Gardner B, Wardle J, Poston L, Croker H (2011). Changing diet and physical activity to reduce gestational weight gain: A meta-analysis. Obes Rev.

[22] Holroyd C, Harvey N, Dennison E, Cooper C (2012). Epigenetic influences in the developmental origins of osteoporosis. Osteoporos Int.

[23] Devlin MJ, Bouxsein ML (2012). Influence of pre- and peri-natal nutrition on skeletal acquisition and maintenance. Bone.

[24] Wood CL, Stenson C, Embleton N (2015). The Developmental Origins of Osteoporosis. Curr Genomics.

[25] Moon RJ, Harvey NC, Cooper C (2015). Endocrinology in pregnancy; Influence of maternal vitamin D status on obstetric outcomes and the fetal skeleton. Eur J Endocrinol.

[26] Ganpule A, Yajnik CS, Fall CH, Rao S, Fisher DJ, Kanade A (2006). Bone mass in Indian children-relationships to maternal nutritional status and diet during pregnancy: the Pune Maternal Nutrition Study. J Clin Endocrinol Metab.

[27] Yin J, Dwyer T, Riley M, Cochrane J, Jones G (2010). The association between maternal diet during pregnancy and bone mass of the children at age 16. Eur J Clin Nutr.

[28] ZA C, Gale CR, Javaid MK, Robinson SM, Law C, Boucher BJ (2009). Maternal dietary patterns during pregnancy and childhood bone mass: a longitudinal study. J Bone Miner Res.

[29] Harvey NC, Javaid MK, Arden NK, Poole JR, Crozier SR, Robinson SM (2010). Maternal predictors of neonatal bone size and geometry: the Southampton Women’s Survey. J Dev Orig Health Dis.

[30] Bisson M, Tremblay F, St-Onge O, Robitaille J, Pronovost E, Simonyan D (2017). Influence of maternal physical activity on infant’s body composition. Pediatr Obes.

[31] Wren TA, Kalkwarf HJ, Zemel BS, Lappe JM, Oberfield S, Shepherd JA (2014). Longitudinal tracking of dual-energy X-ray absorptiometry bone measures over 6 years in children and adolescents: persistence of low bone mass to maturity. J Pediatr.

[32] Harvey N, Dennison E, Cooper C (2014). Osteoporosis: a lifecourse approach. J Bone Min Res..

[33] Liu Z, Qiu L, Chen YM, Su YX (2011). Effect of milk and calcium supplementation on bone density and bone turnover in pregnant Chinese women: a randomized controlled trial. Arch Gynecol Obs.

[34] Chan A-W, Tetzlaff JM, Gøtzsche PC, Altman DG, Mann H, Berlin JA (2013). SPIRIT 2013 explanation and elaboration: guidance for protocols of clinical trials. BMJ.

[35] Davies GA, Wolfe LA, Mottola MF, MacKinnon C (2003). Society of Obstetricians and Gynecologists of Canada, SOGC Clinical Practice Obstetrics Committee. Joint SOGC/CSEP clinical practice guideline: exercise in pregnancy and the postpartum period. Can J Appl Physiol.

[36] Walji R, Wanoush O, Atkinson S (2013). Feasibility and acceptance of a novel nutrition and exercise intervention to manage excess gestational weight gain: focus group study in Ontario, Canada. Prim Heal Care.

[37] Ng M. Maternal vitamin D status during pregnancy as a predictor of offspring bone mass at three years of age (MSc Thesis). Hamilton: McMaster University; 2011.

[38] Josse AR, Atkinson SA, Tarnopolsky MA, Phillips SM (2011). Increased consumption of dairy foods and protein during diet- and exercise-induced weight loss promotes fat mass loss and lean mass gain in overweight and obese premenopausal women. J Nutr.

[39] Mottola MF, Giroux I, Gratton R, Hammond JA, Hanley A, Harris S (2010). Nutrition and exercise prevent excess weight gain in overweight pregnant women. Med Sci Sport Exerc.

[40] Mottola MF (2009). Exercise prescription for overweight and obese women: pregnancy and postpartum. Obstet Gynecol Clin N Am.

[41] Pereira MA, Rifas-Shiman SL, Kleinman KP, Rich-Edwards JW, Peterson KE, Gillman MW (2007). Predictors of change in physical activity during and after pregnancy: Project Viva. Am J Prev Med.

[42] Streuling I, Beyerlein A, Rosenfeld E, Hofmann H, Schulz T, von Kries R (2011). Physical activity and gestational weight gain: a meta-analysis of intervention trials. BJOG.

[43] Institute of Medicine (2002). Dietary Reference Intakes for Energy, Carbohydrate, Fiber, Fat, Fatty acids, Cholesterol, Protein, and Amino Acids (Macronutrients).

[44] Health Canada. Pregnancy weight gain calculator. 2011. http://www.hc-sc.gc.ca/fn-an/nutrition/prenatal/bmi/index-eng.php. Accessed 4 Dec 2018.

[45] Network of Networks Canada. http://n2canada.ca/. Accessed 4 Dec 2018.

[46] Health Canada. Eating Well with Canada’s Food Guide. 2007. http://www.hc-sc.gc.ca/fn-an/food-guide-aliment/index-eng.php. Accessed 4 Dec 2018.

[47] Hymøller L, Jensen SK (2011). Vitamin D analysis in plasma by high performance liquid chromatography (HPLC) with C30 reversed phase column and UV detection - Easy and acetonitrile-free. J Chromatogr A.

[48] Phinney KW, Bedner M, Tai SS, Vamathevan VV, Sander LC, Sharpless KE (2012). Development and certification of a standard reference material for vitamin D metabolites in human serum. Anal Chem.

[49] Winkler TW, Day FR, Croteau-Chonka DC, Wood AR, Locke AE, Mägi R (2014). Quality control and conduct of genome-wide association meta-analyses. Nat Protoc.

[50] Rodrigopulle DJ, Atkinson SA (2014). Validation of surrogate limb analysis for body composition in children by dual energy X-ray absorptiometry (DXA). Eur J Clin Nutr.

[51] Morrison KM, Atkinson SA, Yusuf S, Bourgeois J, McDonald S, McQueen MJ (2009). The Family Atherosclerosis Monitoring In earLY life (FAMILY) study. Rationale, design, and baseline data of a study examining the early determinants of atherosclerosis. Am Heart J.

[52] Harris PA, Taylor R, Thielke R, Payne J, Gonzalez N, Conde JG (2009). Research electronic data capture (REDCap)-A metadata-driven methodology and workflow process for providing translational research informatics support. J Biomed Inform.

[53] Chan AW, Tetzlaff JM, Altman DG, Laupacis A, Gøtzsche PC, Krleža-Jerić K (2013). SPIRIT 2013 statement: Defining standard protocol items for clinical trials. Ann Intern Med.

[54] De Souza RJ, Eisen RB, Perera S, Bantoto B, Bawor M, Dennis BB (2016). Best (but oft-forgotten ) practices : sensitivity analyses in randomized controlled trials. Am Soc Nutr.

[55] Carneiro RM, Prebehalla L, Tedesco MB, Sereika SM, Hugo M, Hollis BW (2010). Lactation and bone turnover: A conundrum of marked bone loss in the setting of coupled bone turnover. J Clin Endocrinol Metab.

[56] Haliloglu B, Ilter E, Aksungar FB, Celik A, Coksuer H, Gunduz T (2011). Bone turnover and maternal 25(OH) vitamin D3 levels during pregnancy and the postpartum period: Should routine vitamin D supplementation be increased in pregnant women?. Eur J Obstet Gynecol Reprod Biol.

[57] van de Lagemaat M, Rotteveel J, Schaafsma A, van Weissenbruch MM, Lafeber HN (2013). Higher vitamin D intake in preterm infants fed an isocaloric, protein- and mineral-enriched postdischarge formula is associated with increased bone accretion. J Nutr.

[58] Young BE, McNanley TJ, Cooper EM, McIntyre AW, Witter F, Harris ZL (2012). Maternal vitamin D status and calcium intake interact to affect fetal skeletal growth in utero in pregnant adolescents. Am J Clin Nutr.

[59] Oken E, Rifas-Shiman SL, Field AE, Lindsay A, Gillman MW (2008). Maternal gestational weight gain and offspring weight in adolescence. Obstet Gynecol.

[60] Boney CM. Metabolic syndrome in childhood: association with birth weight, maternal obesity, and gestational diabetes mellitus. Pediatrics. 2005;115:e290–6 http://pediatrics.aappublications.org/cgi/doi/10.1542/peds.2004-1808. Accessed 4 Dec 2018. 10.1542/peds.2004-1808 15741354

[61] Atkinson SA, Chau K, Ng M (2011). Predictors of post-partum weight retention and adiposity at 3 years in women in Southern Ontario, Canada.

[62] Lowell H, Miller DC (2010). Weight gain during pregnancy: adherence to Health Canada’s guidelines. Health Rep.

[63] Dzakpasu S, Fahey J, Kirby RS, Tough SC, Chalmers B, Heaman MI (2015). Contribution of prepregnancy body mass index and gestational weight gain to adverse neonatal outcomes: Population attributable fractions for Canada. BMC Pregnancy Childbirth.

[64] Subhan FB, Colman I, McCargar L, Bell RC (2017). Higher pre-pregnancy BMI and excessive gestational weight gain are risk factors for rapid weight gain in infants. Matern Child Health J.

[65] Li A, Teo KK, Morrison KM, McDonald SD, Atkinson SA, Anand SS (2017). A genetic link between prepregnancy body mass index, postpartum weight retention, and offspring weight in early childhood. Obesity.

[66] Vasikaran S, Eastell R, Bruyere O, Foldes AJ, Garnero P, Griesmacher A (2011). Markers of bone turnover for the prediction of fracture risk and monitoring of osteoporosis treatment: a need for international reference standards. Osteoporos Int.

[67] Moller UK, Streym S, Mosekilde L, Heickendorff L, Flyvbjerg A, Frystyk J (2013). Changes in calcitropic hormones, bone markers and insulin-like growth factor I (IGF-I) during pregnancy and postpartum: a controlled cohort study. Osteoporos Int.

[68] Hellmeyer L, Ziller V, Anderer G, Ossendorf A, Schmidt S, Hadji P (2006). Biochemical markers of bone turnover during pregnancy: a longitudinal study. Exp Clin Endocrinol Diabetes.

[69] Black AJ, Topping J, Durham B, Farquharson RG, Fraser WD (2000). A detailed assessment of alterations in bone turnover, calcium homeostasis, and bone density in normal pregnancy. J Bone Min Res.

[70] Szulc P, Naylor K, Hoyle NR, Eastell R, Leary ET (2017). Use of CTX-I and PINP as bone turnover markers: National Bone Health Alliance recommendations to standardize sample handling and patient preparation to reduce pre-analytical variability. Osteoporos Int.

[71] Liu NQ, Hewison M (2012). Vitamin D, the placenta and pregnancy. Arch Biochem Biophys.

[72] Brooks SPJ, Greene-Finestone L, Whiting S, Fioletov VE, Laffey P, Petronella N (2017). An analysis of factors associated with 25-hydroxyvitamin D levels in white and non-white Canadians. J AOAC Int.

[73] Wagner CL, Taylor SN, Johnson DD, Hollis BW (2012). The role of vitamin D in pregnancy and lactation: emerging concepts. Women’s Health.

[74] Health Canada. Prenatal nutrition guidelines for health professionals, Background on Canada’s Food Guide. 2009. https://www.canada.ca/en/health-canada/services/food-nutrition/reports-publications/nutrition-healthy-eating/prenatal-nutrition-guidelines-health-professionals-background-canada-food-guide-2009.html. Accessed 4 Dec 2018.

[75] Public Health Agency of Canada. Physical Activity and Pregnancy. 2012. http://www.phac-aspc.gc.ca/hp-gs/guide/assets/pdf/hpguide-eng.pdf. Accessed 4 Dec 2018.

[76] Hardin JW (2001). Generalized Estimating Equations.

[77] Diggle PJ, Liang K-Y, Zeger S (1994). Analysis of longitudinal data.

