# Supplementary material for: Structured diet and exercise guidance in pregnancy to improve health in women and their offspring: study protocol for the Be Healthy in Pregnancy (BHIP) randomized controlled trial
Source: Trials. 2018 Dec 19;19:691. doi: 10.1186/s13063-018-3065-x (PMC6299965; doi:10.1186/s13063-018-3065-x)
Supplement: Supplementary file 2 — WHO Trial registration data set. (DOCX 18 kb) [file 13063_2018_3065_MOESM2_ESM.docx]

**Additional file 2:** Items from the World Health Organization Trial Registration Data Set

| **Item** | **Description** |
| --- | --- |
| 1. Primary registry and trial-identifying number | Primary Registry: ClinicalTrials.gov  Identifying Number: NCT01689961 |
| 2. Date of registration in primary registry | September 13, 2012 and September 20, 2012 |
| 3. Secondary identifying numbers | n/a |
| 4. Sources of monetary or material support | Monetary Support   1. Canadian Institutes of Health Research (CIHR) (FRN 123347) 2. Dairy Research Cluster Initiative (Dairy Farmers of Canada and Agriculture and Agri-Food Canada (AAFC), the Dairy Network and the Canadian Dairy Commission)   In-kind donation   1. GayLea Foods Coop, Canada 2. Ultima Foods, Canada |
| 5. Primary Sponsor | Canadian Institutes of Health Research (CIHR) (FRN 123347) |
| 6. Secondary Sponsor | Dairy Research Cluster Initiative (Dairy Farmers of Canada and Agriculture and Agri-Food Canada (AAFC), the Dairy Network and the Canadian Dairy Commission) |
| 7. Contact for Public Queries | Dr. Stephanie A. Atkinson  Dept Pediatrics, HSC 3A44, McMaster University  1280 Main St W  Hamilton, ON, Canada L8S 4K1  Email: [satkins@mcmaster.ca](mailto:satkins@mcmaster.ca) |
| 8. Contact for Scientific Queries | Dr. Stephanie A. Atkinson  Dept Pediatrics, HSC 3A44, McMaster University  1280 Main St W  Hamilton, ON, Canada L8S 4K1  Email: [satkins@mcmaster.ca](mailto:satkins@mcmaster.ca) |
| 9. Public title | BHIP study |
| 10. Scientific title | Structured diet and exercise guidance in pregnancy to improve health in women and their offspring: study protocol for the *Be Healthy in Pregnancy (BHIP)* randomized controlled trial |
| 11. Countries of recruitment | Canada |
| 12. Health condition(s) or problem(s) studied | Gestational weight gain  Developmental origins of health and disease – Bone metabolism |
| 13. Intervention(s) | Maternal Nutrition + Exercise intervention from 17 to 38 week gestation |
| 14. Key inclusion and exclusion criteria | **Inclusion Criteria**: Healthy pregnant females > 18 years of age with singleton pregnancies (either nulliparous or multiparous); may be randomized to group allocation by 17 weeks and 6 days gestation; a pre-pregnancy BMI < 40 kg/m^2^; plans to deliver at a Hamilton, Burlington or London regional hospital or by home birth but willing to attend research visits at either study site; approval of primary care provider to exercise; and able to provide signed informed consent.  **Exclusion Criteria:** Not conversant in English; known contraindications to exercise as recommended by the Canadian clinical practice guidelines for pregnancy; severe chronic gastrointestinal, heart, kidney, liver or pancreatic diseases or conditions; refusal to consume dairy foods due to intolerance or dislike; pre-existing diabetes; currently smoking and will not discontinue smoking during the pregnancy; or a depression score above 12 on the validated Edinburgh Depression scale. |
| 15. Study type | **Allocation**: Randomized  **Blinding:** Investigators and Research Staff are not blinded to participant assignment. Primary outcome assessors and statisticians are blinded to assignment  **Phase**: Ongoing  **Method of Sequence Generation:** Computer Generated Allocation sequence with stratification by study site and BMI, and block of 2, 4, or 6 at random  **Method of Allocation Concealment:** Use of a Third party randomization technique |
| 16. Date of First Enrolment | January 2013 |
| 17. Target Sample Size | 350 participants |
| 18. Recruitment Status | Enrolling |
| 19 Primary Outcome | Gestational weight gain |
| 20. Key Secondary Outcomes(s)  Clinical Outcomes | **Bone biomarkers in maternal and cord blood**: Procollagen type I that contains N-terminal extensions (PINP), C-terminal telopeptide of type I collagen (CTX-I), Insulin-like growth factor (IGF-1), Vitamin D metabolites  **Maternal bone status**: whole body bone mineral content (BMC) and whole body BMD, lumbar spine BMD  **Infant bone status:** Infant’s bone health status at 6 months of age measured by Dual energy x-ray absorptiometry scan (DXA), and expressed as whole body minus the head BMC and BMD |
